# Preclinical Evaluation of a ^64^Cu-Based Theranostic Approach in a Murine Model of Multiple Myeloma

**DOI:** 10.3390/pharmaceutics15071817

**Published:** 2023-06-25

**Authors:** Cassandra Métivier, Patricia Le Saëc, Joëlle Gaschet, Catherine Chauvet, Séverine Marionneau-Lambot, Peter O. Hofgaard, Bjarne Bogen, Julie Pineau, Nathalie Le Bris, Raphaël Tripier, Cyrille Alliot, Férid Haddad, Michel Chérel, Nicolas Chouin, Alain Faivre-Chauvet, Latifa Rbah-Vidal

**Affiliations:** 1 Nantes Université, Inserm, CNRS, Université d’Angers, CRCI^2^NA, 44007 Nantes, France; cassandra.metivier@etu.univ-nantes.fr (C.M.); patricia.lesaec@univ-nantes.fr (P.L.S.); joelle.gaschet@univ-nantes.fr (J.G.); catherine.chauvet@univ-nantes.fr (C.C.); cyrille.alliot@univ-nantes.fr (C.A.); michel.cherel@univ-nantes.fr (M.C.); 2 Nantes Université, Inserm, CNRS, Université d’Angers, CRCI^2^NA, CHU Nantes, 44007 Nantes, France; severine.marionneau-lambot@univ-nantes.fr (S.M.-L.); alain.faivre-chauvet@univ-nantes.fr (A.F.-C.); 3 Department of Immunology, Oslo University Hospital, 04024 Oslo, Norway; p.o.hofgaard@medisin.uio.no (P.O.H.); bjarne.bogen@medisin.uio.no (B.B.); 4 Univ. Brest, UMR CNRS 6521 CEMCA, 6 Avenue Victor le Gorgeu, 29200 Brest, France; julie.pineau@univ-brest.fr (J.P.); nathalie.lebris@univ-brest.fr (N.L.B.); raphael.tripier@univ-brest.fr (R.T.); 5 GIP ARRONAX, 44800 Saint-Herblain, France; 6 IMT Atlantique, Nantes Université, Subatech, 44307 Nantes, France; haddad@arronax-nantes.fr; 7 Nuclear Medicine Department, ICO-René Gauducheau Cancer Center, 44800 Saint-Herblain, France; 8 Nantes Université, Inserm, CNRS, Université d’Angers, Oniris, CRCI^2^NA, 44007 Nantes, France; nicolas.chouin@oniris-nantes.fr

**Keywords:** theranostics, copper-64, PET imaging, targeted radionuclide therapy, dosimetry, CD138, multiple myeloma

## Abstract

Although the concept of theranostics is neither new nor exclusive to nuclear medicine, it is a particularly promising approach for the future of nuclear oncology. This approach is based on the use of molecules targeting specific biomarkers in the tumour or its microenvironment, associated with optimal radionuclides which, depending on their emission properties, allow the combination of diagnosis by molecular imaging and targeted radionuclide therapy (TRT). Copper-64 has suitable decay properties (both β^+^ and β- decays) for PET imaging and potentially for TRT, making it both an imaging and therapy agent. We developed and evaluated a theranostic approach using a copper-64 radiolabelled anti-CD138 antibody, [^64^Cu]Cu-TE1PA-9E7.4 in a MOPC315.BM mouse model of multiple myeloma. PET imaging using [^64^Cu]Cu-TE1PA-9E7.4 allows for high-resolution PET images. Dosimetric estimation from ex vivo biodistribution data revealed acceptable delivered doses to healthy organs and tissues, and a very encouraging tumour absorbed dose for TRT applications. Therapeutic efficacy resulting in delayed tumour growth and increased survival without inducing major or irreversible toxicity has been observed with 2 doses of 35 MBq administered at a 2-week interval. Repeated injections of [^64^Cu]Cu-TE1PA-9E7.4 are safe and can be effective for TRT application in this syngeneic preclinical model of MM.

## 1. Introduction

Precision medicine is fundamentally based on the accurate and specific targeting of cancer cells. This involves, where possible, patient selection based on biological or molecular imaging tests that subsequently allow eligible patients to be offered targeted therapy. This selection is necessary in order to secure patients that are more likely to respond and therefore avoid the use of expensive treatments on patients who are unlikely to respond. The impressive evolution of diagnostic technologies, combined with the advent of targeted therapies, has largely contributed to the development of the concept of theranostics, which integrates diagnostic tools with therapeutic strategies and has become mainstream in personalized medicine.

In nuclear medicine, the concept of theranostics is based on the use of a single or two very similar radiopharmaceuticals named a “theranostic pair” for diagnosis and therapy. Ideally, this consists of a chemically and structurally identical (or nearly identical) vector labelled with a diagnostic or therapeutic radionuclide [1]. In some cases, the radionuclide may itself be both a diagnostic and a therapeutic agent. Indeed, some radionuclides have this particularity, including lutetium-177 (^177^Lu; β- emitter) and copper-67 (^67^Cu; β- decay and γ rays), or copper-64 (^64^Cu; both β^+^ and β- decays) and ^149^Tb (both β^+^ and α decays).

In recent years, several studies have shown an interest in evaluating the theranostic potential of copper-64 in cancer therapy. This interest is motivated by the particular decay properties of ^64^Cu, which make it an almost perfect example of a theranostic radionuclide [2,3,4]. Indeed, ^64^Cu emits both low-energy positrons, allowing one to obtain high resolution PET images when using low activity, and β- particles and Auger electrons that allow, via adaptation of the administered activity, for the obtaining of a therapeutic effect.

In 2001 [5], [^64^Cu]Cu-ATSM was evaluated as a treatment in a human colorectal tumour model transplanted in hamsters. Escalation of activity involving i.p. administration of increasing activities of [^64^Cu]Cu-ATSM allowed identification of the effective activity margin (222 MBq < effective activity < 370 MBq) that induced a significant improvement in survival. The interest of dose fractionation was also evaluated (3 × 148 MBq at 3 days interval). In parallel, a 24-h PET imaging follow-up of [^64^Cu]Cu-ATSM allowed for the monitoring of the evolution of the volume and the morphology of the tumours and the estimation of the dosimetry at the tumour.

More recently, Yoshii et al. [6] showed that intraperitoneal administration of 22.2 MBq of [^64^Cu]Cu-PCTA-cetuximab during the early phase of administration disease development prolonged the survival of mice without inducing acute toxicity, while treatment during the late phase of disease development allowed PET imaging-guided microsurgery of disseminated tumours. These results show that the same radiopharmaceutical was firstly used to localize the tumour lesions and to guide ablative surgery, and secondly to confirm the absence of tumour residues after surgery. Some other preclinical studies have demonstrated the therapeutic properties of copper-64. In one of the first studies, published over 20 years ago [7], a copper-64-labelled somatostatin analogue, [^64^Cu]Cu TETA-Tyr3-Octreotate was evaluated in a rat model of pancreatic cancer. The results show a significant slowing of tumour growth following a single dose injection (573.5 MBq) and a complete arrest of tumour growth for 10 days, before tumour regrowth with repeated doses (3 × 740 MBq at 2-day intervals). This treatment efficacy was accompanied by transient haematological and hepatic toxicity.

Beyond these promising results, other, more recent studies, have shown that copper-64 based therapy was only effective in combination with other anti-cancer treatments [8] or as adjuvant therapy [9]. In another study, two therapy experiments were performed simultaneously in a mouse model of glioblastoma s.c, with [^64^Cu]Cu-RaftRGD, a peptide targeting integrins found in tumour neovessels, and [^64^Cu]Cu-ATSM, which accumulates specifically in hypoxic areas [10]. Intravenous administration of [^64^Cu]Cu-RaftRGD with [^64^Cu]Cu-ATSM (18.5 MBq each) significantly increased the median survival of mice compared with the untreated group, whereas injection of 37 MBq of either treatment alone had no effect on improving median survival. In this study, it is the improved distribution of copper-64 within the tumour, via the targeting of two different aspects of its phenotype, that increases therapeutic efficacy.

Very recently, a vector targeting PSMA, [^64^Cu]Cu-DOTAH2-PSMA, was evaluated for theranostic applications in mice bearing LNCaP prostate tumours, with very promising results for both imaging [11] and therapy [12]. PET imaging showed optimal tumour uptake at 4 h p.i. and evaluation of therapeutic potential showed that a single dose of 150 MBq [^64^Cu]Cu-DOTAH2-PSMA or 120 MBq [^177^Lu]Lu-PSMA-617 significantly increased the median survival of mice compared with control mice in a similar manner and without inducing significant toxicity.

Herein we aimed to evaluate a theranostic approach combining the advantageous properties of copper-64 and the high specificity of antibodies (radioimmunotheranostics) using the anti-CD138 monoclonal antibody 9E7.4 in an aggressive mouse model of multiple myeloma.

The first characterisation and validation of this monoclonal antibody (mAb) was published by our research team in 2015 [13]. 9E7.4 is a rat IgG2a,κ type immunoglobulin that reacts specifically with the extra-membrane part of mouse syndecan-1 (CD138) and with a very good affinity of the order of 10^−10^ M. 9E7.4 was evaluated in a targeted radionuclide therapy in immunocompetent mice bearing 5T33-multiple myeloma tumours. Labelled with astatine-211 [14] and bismuth-213 [13], two alpha-emitters, it showed a very good efficacy with, respectively, 65% and 45% of mice surviving on a long-term basis. Results observed with lutetium-177 [13] were less spectacular: a single dose of β-RIT was not sufficient to cure mice developing MM. 9E7.4 has also been tested as an imaging agent after radiolabelling with copper-64 [15], and radiolabelling with zirconium-89 [16]. The results of these two studies demonstrate the ability of ^64^Cu-radiolabelled anti-CD138 (9E7.4) to detect subcutaneous MM tumours and bone marrow lesions with high sensitivity, outperforming [^18^F]-2-FDG. Additionally, imaging with 9E7.4, radiolabelled with copper-64, seems to be a better choice than imaging with 9E7.4 radiolabelled with zirconium-89, probably due to the new copper chelating agents that provide better protection against the transchelation phenomenon. Similar results are described in literature with other antibodies and/or radionuclides. A recent review, published in 2021 [17], also describes the interest of radiolabelled antibodies to increase the tumour dose link with its long biologic half-life. This property is particularly interesting in regard to haematologic tumours that are easily accessible via the bloodstream. 

Therefore, considering this property and assuming that the biodistribution of the mAbs-based radiotracers is representative of the biodistribution of therapeutic mAbs, in the present work we investigate the ability of 9E7.4 radiolabelled with coopper-64 to specifically visualize and treat CD138-expressing myeloma tumours.

## 2. Materials and Methods

### 2.1. Cells and Reagents

The MOPC315.BM murine MM cell line was provided by Pr Bjarne Bogen (Department of Immunology, Centre for Immune Regulation, Institute of Clinical Medicine, University of Oslo, Oslo, Norway) [18]. The cells were cultured in RMPI medium (Gibco/Invitrogen, Saint Aubin, France) containing 2 mM l-glutamine, 100 U/mL penicillin/100 µg/mL streptomycin, 1% MEM NEAA 100× (Gibco), 1% sodium pyruvate (Gibco), and 10% heat-inactivated fetal calf serum (Corning, Thermo Fisher Scientific, Villebon-sur-Yvette, France). The 5T33 murine MM cell line was provided by Dr Harvey Turner (Department of Nuclear Medicine, Fremantle Hospital, Fremantale, Western Australia, Australia) with permission from Dr J. Radl (TNO Institute, Leiden, The Netherlands). 5T33 cells were cultured in RPMI 1640 medium (Life Technologies) containing 2 mM l-glutamine, 100 U/mL penicillin/100 µg/mL streptomycin and 10% heat-inactivated fetal calf serum (Corning). The cells were passed 3 times per week and maintained at 37 °C under 5% CO_2_ and 95% humidity. Cells were used for experiments at low confluence in the exponential phase and a passage lower than P12. The 9E7.4 mAb was produced by immunization of a rat with a 40-amino-acid peptide (GeneCust) derived from the murine CD138 protein (aa 90-130) (GenBank: CAA80254.1). The specificity and affinity of 9E7.4 mAb for CD138 and the expression of the target by MOPC315.BM cells were determined by flow cytometry. Staining was acquired on FACS Calibur™ using Diva software (Version6.0, BD Biosciences, San Jose, CA, USA) and then analysed on FlowJo™ software (version 10.6, BD Biosciences).

### 2.2. Animal Model

All animal experiments were carried out in accordance with the European directive 2010/63/EU on the protection of laboratory animals and its transposition into French law (decree n°2013-118). The preclinical studies were conducted on the UTE platform (SFR François Bonamy, IRS-UN, University of Nantes) (authorisation D44-278). The authorisation for the animal experimentation project (APAFIS #22766 for targeting radiotherapy experiments, APAFIS #6145 for targeting experiments) was delivered by the Ministry of Higher Education and Research, after a favourable review from the local animal experimentation ethics committee “Pays de Loire (CEEA-06)”. For all our in vivo experiments, we used BALB/cJRj immunocompetent female mice purchased from Janvier laboratories. These were housed in the UTE animal house, in ventilated cages (5 mice per cage) under sterile and pathogen-free conditions and with free access to food and water. Mice were 8–11 weeks old at the time of experiments and had an average weight of 19–20 g. After reception, mice were acclimated for 7 days before any experimental manipulations. Experiments were carried out on an orthotopic model established by an i.v injection of 2 × 10^5^ cells in 100 µL of 0.9% NaCl into the tail vein and a subcutaneous allograft model established by subcutaneous injection of 2 × 10^5^ cells in 100 µL of 0.9% NaCl on the right shoulder of mice under anaesthesia with a mixture of 3% isoflurane and oxygen. Before each engraftment of the tumour cells, the absence of mycoplasma contamination of the cells was checked using a HEK-blue detection kit (Invivogen, Toulouse, France). The orthotopic model grew for 9 days and the subcutaneous tumours grew for 6 days before subsequent studies.

### 2.3. Anti-CD138 mAb Conjugation and Radiolabelling

9E7.4-p-SCN-Bn-TE1PA was obtained with a standard conjugation method [19,20]. The batch of conjugated antibody used for radioimmunotherapy experiments was coupled with 4 chelates per mAb. Copper-64 dichloride in 0.1 M hydrochloric acid solution ([^64^Cu]CuCl_2_) was provided by ARRONAX cyclotron (Saint-Herblain, France). Radionuclide purity was determined by gamma spectrometry using a DSPEC-JR-2.0 type 98–24B HPGE detector (AMETEK). Chemical purity was evaluated by ICP-OES using an iCAP 6500 DUO (Thermo Fischer Scientific, Waltham, MA, USA) (Table S1). Ten percent of sodium acetate (2.5 M) was added to [^64^Cu]CuCl_2_ volume to maintain a pH of 6. According to the quantities of metals present per source received, a calculated quantity of 9E7.4-TE1PA was added to copper acetate solution in order to obtain an antibody-to-total metals ratio of close to 1 to avoid purification after radiolabelling. The reaction mixture volumes were adjusted with 0.1 M sodium acetate and then incubated at 42 °C for 30 min. Ten antibody equivalents of EDTA 1 mM were added to stop the reaction and complex-free ^64^Cu(II). Volume adjustment for animal injections was undertaken with 0.9% NaCl. One µL of the radiolabelling solution was deposited on an instant thin layer chromatography plate (Agilent Technologies, Lake Forest, CA USA) to evaluate the radiochemical purity. The ITLC was performed using citrate buffer (0.1 M; pH 4.5). After migration, the plate was exposed on a storage phosphor plate read by a radiometric phosphor imager Cyclone Plus (PerkinElmer, Shelton, CT, USA) and analysed by OptiQuant software (Version 5).

An immunoreactivity test was performed after each radiolabelling. NHS-activated magnetic beads (ThermoFischer Scientific) were previously conjugated with a CD138-like peptide provided by Genecust using the protocol of the producer. Conjugated beads were placed in PBS at a final concentration of 10 mg/mL. Ten µL of the beads suspension was added to 1/0.5/0.2 pmol of [^64^Cu]Cu-TE1PA-9E7.4. A PBS/BSA 0.1% solution was added to samples to a 100 µL final volume. Samples were incubated at room temperature during 30 min on a rotator. Using a magnetic rack (MagRack 6, GE Healthcare, Uppsala, Sweden), supernatants and beads were collected separately and counted with a gamma counter (Wallac 1480 Wizard γ-counter, PerkinElmer). The immunoreactivity was calculated as the percentage of the number of counts per minute (CPM) of the reactive radiolabelled antibodies on beads compared with the total activity including supernatant and beads.

### 2.4. Cellular Uptake

Two days prior to the experiments, cells were plated on 6-well culture plates at a concentration of 1 × 10^5^ MOPC315.BM cells/mL per well. Then, 334 pmol of 9E7.4- TE1PA and BSA- TE1PA were radiolabelled with [^64^Cu]CuCl_2_. Cells were incubated with increasing concentrations of [^64^Cu]Cu-TE1PA-9E7.4 (4 datapoints: 0.5, 1, 1.8 and 5.5 pmol/well) alone, with an excess of at least 100 equivalent of 9E7.4 mAb, or with similarly increasing concentrations of [^64^Cu]Cu-TE1PA-BSA as a negative control. After 1, 24 and 48 h of incubation at 37 °C cells were counted under Malassey using eosin, washed twice and isolated from the supernatant by centrifugation at 1500 rpm for 5 min. Cells and supernatants radioactivities were counted using an automatic gamma counter, Hidex AMG (HIDEX, Finland). The cellular uptake was expressed as the percentage of deposed activity per millions of cells. Statistical analysis was undertaken using an unpaired *t* test at 5% significance level.

### 2.5. Ex Vivo Biodistribution

Two ex vivo biodistribution experiments (21 mice/experiment) were performed in the same conditions on normal (tumour-free) mice and on mice bearing s.c tumours (306 ± 95.8 mm^3^). Mice had received 8.5 ± 0.6 MBq of 25 µg of [^64^Cu]Cu-TE1PA-9E7.4 via the tail vein. Mice were sacrificed at 5 min, 30 min, 3 h, 24 h, 48 h (4 mice/group) and 72 h post-injection (5 mice). Tumours, blood and organs were harvested, weighed on a precision balance (Adventurer™ Precision, OHAUS) and radioactivity was counted using an automatic gamma counter, Hidex AMG (HIDEX, Finland). Counter efficacy was evaluated using standard references (*n* = 10). To refine the analysis, the injected activity per mouse was corrected by removing the activity remaining at the injection site in the tail. Results were decay corrected at injection time and expressed as a percentage of injected dose per gram of tissue. Statistical analysis was undertaken using an unpaired *t* test at 5% significance level.

### 2.6. Positron Emission Tomography (PET) Imaging

Mice were imaged at 5 min, 24 h, 48 h and 72 h p.i. A group of mice imaged at 72 h p.i. was used for the 72 h p.i. time of biodistributions. Mice were maintained under general anaesthesia with a mixture of 2% isoflurane and 5% air for the duration of the acquisition. Image acquisition was performed with the IRIS PET-CT imager (Iris, Inviscan, Strasbourg France) using a 10-min static whole-body acquisition mode combined with a 20-s CT acquisition (energy window of 250-750 keV and FOV size of 90 mm). The CT acquisition parameters were 80 kV, 0.9 mA, 576 projections and 160 µm voxel size. Filtered back-projection algorithm with beam hardening and ring artefact correction was used. PET images were reconstructed with the 3D Ordered Subsets Expectation Maximization (3D-OSEM-MC,) algorithm, 8 subsets, 8 iterations with correction of decay, random and dead-time. PET and CT were converted into DICOM format and processed with OsiriX Medical Imaging software (version 11.0.1, Pixmeo, Bernex, Switzerland).

### 2.7. Dosimetry

Dosimetry was performed based on ex vivo biodistribution study results. Mean absorbed doses to the main tissues/organs and tumour were calculated following the MIRD scheme [21]. Time–activity curves for each organ/tissue were derived from the ex vivo biodistributions and fitted with a single exponential function or a sum of two exponentials using GraphPad PRISM 7 (GraphPad Software Inc., San Diego, CA, USA). The cumulated activities in each organ/tissue were calculated by integrating these single or dual exponential functions between 0 and infinity. For most tissues, self-dose and cross-dose S-values were calculated with the GATE (v 9.0) Monte Carlo code [22] using a realistic mouse phantom Moby of 20 g [23]. For tumours, salivary glands and ganglia, self-dose S-values were calculated with the IDAC-Dose 2.1 software [24] assuming these tissues have a spherical shape. Mean absorbed dose to blood was estimated assuming that all emitted energy in the blood was absorbed locally.

### 2.8. Therapeutic Studies

In the orthotopic model, a dose-escalation study was performed with a [^64^Cu]Cu-TE1PA-9E7.4 single injection of 22 (*n* = 10), 35 (*n* = 10), 55 (*n* = 5) or 65 MBq (*n* = 7). Then, a [^64^Cu]Cu-TE1PA-9E7.4 dose repetition of 35 (*n* = 6) and 55 MBq (*n* = 6) was undertaken at a 2-week interval. Control groups have received i.v injection of saline solution (*n* = 22), 9E7.4 mAb (*n* = 10), or 35 MBq of [^64^Cu]CuCl_2_ (*n* = 5). In the subcutaneous model, a dose repetition of saline solution (*n* = 15), 35 MBq of [^64^Cu]CuCl_2_ (*n* = 3) or 35 MBq of [^64^Cu]Cu-TE1PA-9E7.4 (*n* = 9) was undertaken with a 2-week dose interval. After treatment, survival was monitored over time. Mice were sacrificed when paralysis or extramedullary tumours were detected, when 20% of their body weight was lost or if there were other signs of significant toxicity. In the s.c model, tumour growth was measured by the same operator using a digital calliper, the volume was calculated using the formula: volume = (L × W × h) × 3.14/6, where L was length, W was width and h was height [25]. Mice were sacrificed when tumour volume reached 2000 mm^3^ or at the apparition of necrosis in tumours or at any sign of pain and/or toxicity. Statistical analysis of tumour volumes was performed using a two-way ANOVA followed by Sidak’s multiple comparison test.

### 2.9. Toxicity Monitoring

Haematological toxicity was monitored weekly after tumour transplantation, with white blood cell, platelet, and erythrocyte counts using an automated quantitative haematological analyser with 25 µL of blood per test (Celltac-α mek-6500k, Nikon Kohden, Rosbech, Germany). Blood samples were collected from the mouse retroorbital sinus under general gas anaesthesia with 2.5% isoflurane and 5% air and collected in tubes containing EDTA (Laboratoires Melet Schlloesing, Osny, France). Blood samples were centrifuged at 2500 rpm for 10 min in order to recover the plasma, which was stored at −20 °C until it was analysed for liver and kidney toxicity. Plasma was used to assess liver and kidney toxicity by measuring the following biochemical parameters: aspartate aminotransferase (AST) and alanine aminotransferase (ALT) to monitor liver status, and blood urea nitrogen (BUN) and creatinine to measure renal toxicity. First, plasma samples were thawed on ice and then diluted or undiluted according to their initial volume with 0.9% NaCl to obtain a final volume of 100 µL required for analysis. Dilutions up to a maximum of ¼ were made in order not to reach the detection limit of the device. Plasma from each of the mice was then placed in a rotor (General Health Rotor, Scil) containing the reagents required for the various tests and processed using a fully automated biochemistry analyser (Element RC, Scil animal care company, Altorf, France) allowing the measurement of parameters using photometric tests. A cohort of healthy BALB/cJRj mice was used to calculate the reference values of the studied parameters. Statistical analysis was performed using a two-factor ANOVA followed by a Sidak’s multiple comparison test.

### 2.10. Histolgy

Tissues were removed, fixed in formalin and transferred to 70% ethanol before being embedded in paraffin. Paraffin sections of 2 μm were cut and the tissue sections were mounted on Trajan frosted™ slides. Slices were stained with Hematoxylin Phoxine-Safran by Cellular and Tissular Imaging Core Facility of Nantes University (MicroPICell, Nantes, France). A Nanozoomer Hamamatsu™ slide scanner was used to scan the slides.

## 3. Results 

### 3.1. Comparison of CD138 Expression on MOPC315.BM and 5T33 Cells

**Figure 1:**
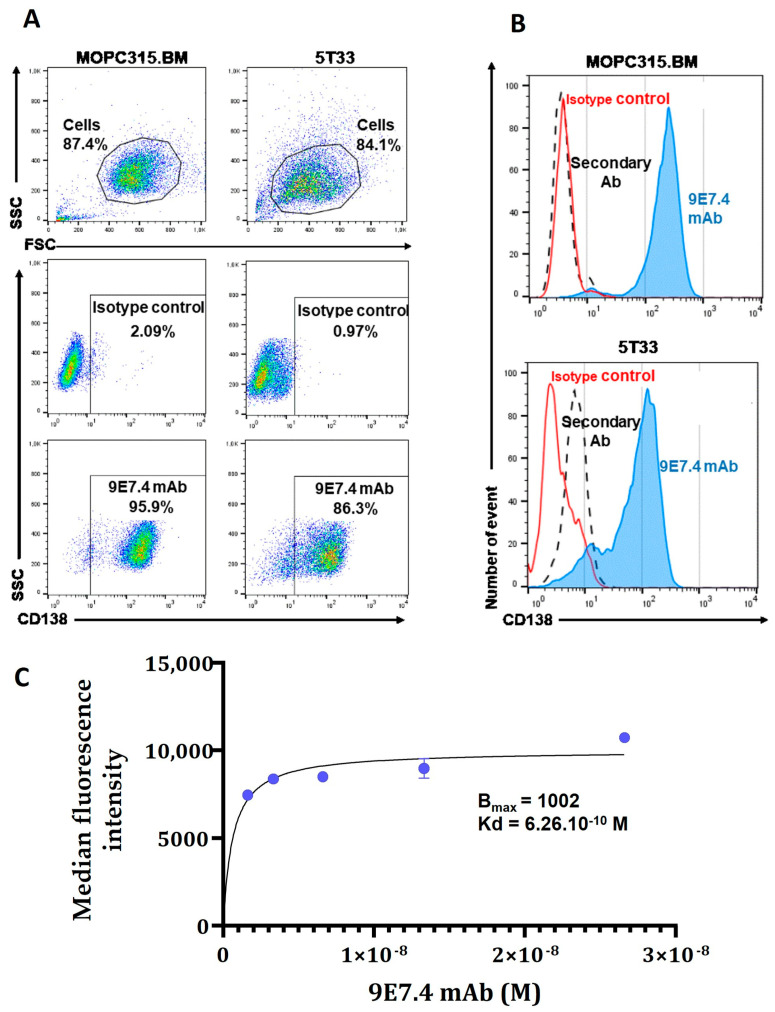
Characterization of in vitro CD138 membrane expression in 5T33 and MOPC315.BM murine cell lines by flow cytometry. (**A**) Cell analysis strategy: cells were selected based on their size (FSC parameter) and granulation (SSC parameter) to exclude debris and dead cells. Cell labelling was performed with 9E7.4 anti-CD138 antibody and IgG2a isotype at a saturating concentration of 25 µg/mL. (**B**) Representative histograms of phycoerythrin (PE) fluorescence intensity of control isotype (red curve) or 9E7.4 antibody (blue curve) and secondary antibody (black dashed curve). Acquisition was performed on a BD FACS Calibur™ flow cytometry system and analysed using FlowJo™ software -V10.6™ (BD Biosciences). (**C**) Binding saturation curve for 9E7.4 as determined using flow cytometry after incubation of MOPC315.BM cells with increasing antibody concentrations (from 4 µg/mL to 0.25 µg/mL). Antibody affinity analysis was performed on GraphPad Prism software (version 8.0.0 for Windows, GraphPad Software, San Diego, CA, USA) using a nonlinear regression curve (“One site-Specific binding”). Error bars represent the standard deviation of the fluorescence medians.

The specificity of mAb 9E7.4 for murine CD138 has been confirmed previously in the 5T33 cell line [13]. Here, we evaluated, by flow cytometry, the expression of CD138 in MOPC315.BM compared with the 5T33 cell line (Figure 1). The results show strong CD138 expression in both MM cell lines, with good specificity confirmed by the absence of non-specific binding of the control isotype (Figure 1A). However, CD138 expression appears slightly higher and more homogeneous (Figure 1B) in MOPC315.BM cells (95.9% versus 86.3% in 5T33 cells). Based on these results, MOPC315.BM cells were selected for the next steps of this work. Furthermore, the MOPC315.BM line is implantable in immunocompetent mice with the occurrence of paraplegia (sign of spinal cord compression) in all mice within 3–4 weeks [18] and has tropism for the bone marrow, inducing osteolytic lesions that mimic human disease. Thereafter, the affinity of the 9E7.4 antibody for the CD138 target on the MOPC315.BM cell line was determined by flow cytometry with increasing concentrations of the antibody (Figure 1C). Results demonstrate a dissociation constant (Kd) value of 0.626 nM. 

### 3.2. Radiolabelling and Immunoreactivity

The chemical purities of the copper-64 sources used in this study are between 21 and 63 MBq/nmole, considering all metallic contaminants research by ICP-OES (Copper, Zinc, Cobalt, Iron, and Nickel) has a specific activity above 100 MBq/nmole. Synthesis and radiolabelling were performed using previously published procedures [26]. Note that in this article, the radioimmunoconjugate [^64^Cu][Cu-9E7.4-(p-SCN-Bn-TE1PA)] has been named [^64^Cu]Cu-TE1PA-9E7.4 for simplification. For all radiolabelling experiments, the radiochemical purity of [^64^Cu]Cu-TE1PA-9E7.4 was greater than 98% and radiolabelling yield was from 90 to 100%. Specific activity of [^64^Cu]Cu-TE1PA-9E7.4 was different for each cohort of mice because we chose to inject a fixed amount of antibody per mouse (25 µg for the PET images and 100 µg for the therapies). Thus, specific activity of each cohort, ranging a minimum of 236 MBq/mg to a maximum of 651 MBq/mg, is reported in Table S1. After radiolabelling, integrity of the 9E7.4 antibody was verified by estimating its immunoreactive fraction using magnetic beads coated with CD138 antigen. This control was performed after each radiolabelling and confirmed an immunoreactivity of 79.4% ± 0.8%.

### 3.3. In Vitro Cellular Binding 

**Figure 2:**
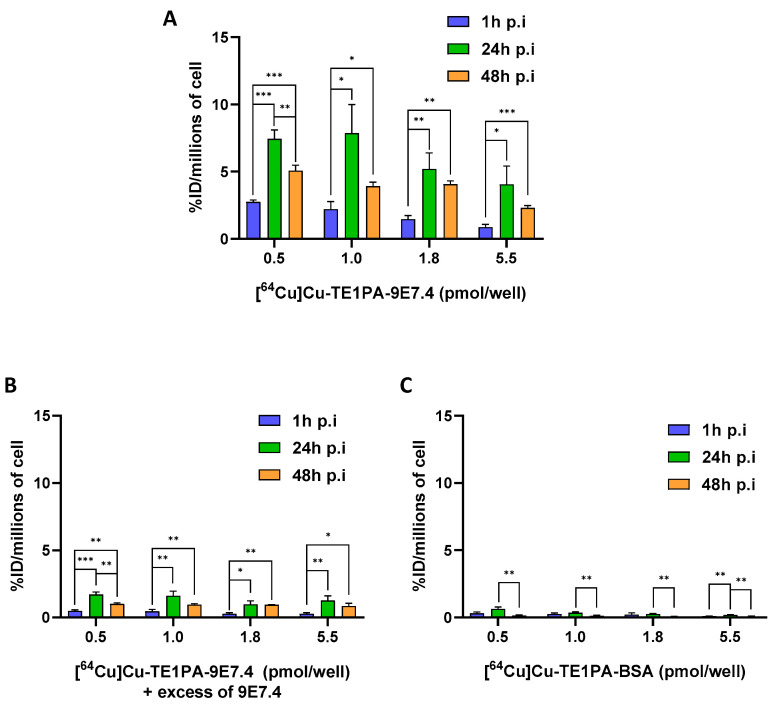
In vitro cellular binding assays. Cellular binding of (**A**) [^64^Cu]Cu-TE1PA-9E7.4, (**B**) [^64^Cu]Cu-TE1PA-9E7.4 with an excess of 9E7.4, (**C**) [^64^Cu]Cu-TE1PA-BSA, to MOPC315.BM cells using 4 concentrations of radiotracers at 3 different time post-incubation. Results are expressed as % activity injected per million cells (*n* = 3; multiple *t*-test, (*) *p* < 0.02, (**) *p* < 0.009, (***) *p* < 0.0007).

In vitro MOPC315.BM cellular uptake of [^64^Cu]Cu-TE1PA-9E7.4 was assessed at 37 °C for 1, 24, and 48 h of incubation of cells with increasing concentrations of the radiotracer (Figure 2). After 1 h incubation at 37 °C, [^64^Cu]Cu-TE1PA-9E7.4 binding was significant for all concentration conditions, with a maximum uptake of 2.8 ± 0.1 %ID/millions of cells for 0.5 pmol/well concentration and a minimum of 0.887 ± 0.14 %ID/millions of cells for 5.5 pmol/well concentration. After 24 h, maximum uptake of the compound on cells was reached independently of concentration used. Uptake was significantly higher than at 1 h of incubation, with a maximum of 7.9 ± 1.5 %ID/million cells for 1 pmol/well (*p* = 0.01). At 48 h, we observed a considerable decrease in tracer binding compared with 24 h. However, it remained significantly higher than the uptake at 1 h of incubation. For example, with 1.8 pmol/well, a significant increase in uptake is observed (from 1.47 ± 0.19 %ID/million cells at 1 h to 4.1 ± 0.2 %ID/million cells after 48h of incubation, *p* = 0.0017)). 

Binding of [^64^Cu]Cu-TE1PA-9E7.4 was significantly blocked (>75% at 24 h) by coincubation with an excess of non-radiolabelled 9E7.4 antibody (Figure 2B) demonstrating the CD138 specificity of the radioimmunoconjugate. An additional control experiment was performed using [^64^Cu]Cu-TE1PA-BSA (Figure 2C). For the negative control with [^64^Cu]Cu-TE1PA-BSA, no condition exceeded the 0.6 ± 0.1% ID/million cell uptake obtained with the 0.5 pmol/well concentration at 24 h incubation.

### 3.4. Ex Vivo Biodistribution and PET-CT Imaging 

**Figure 3:**
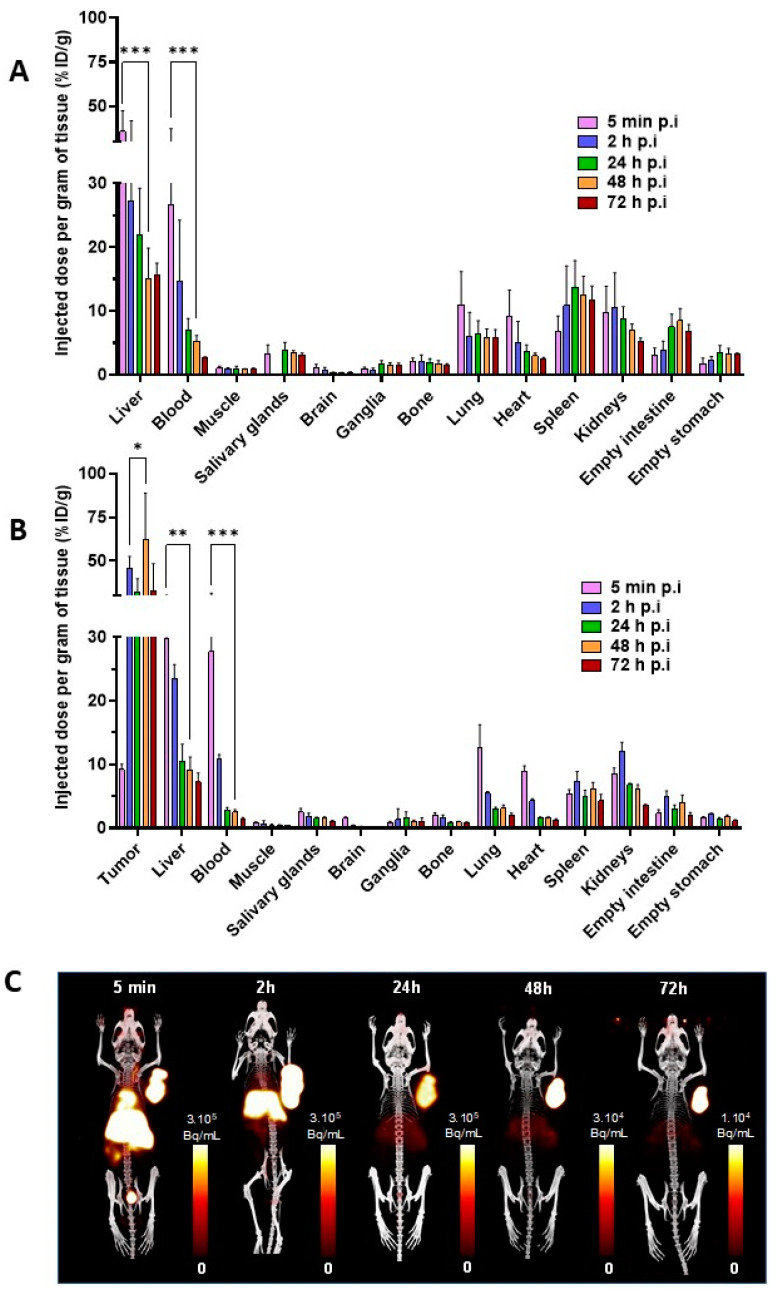
Ex vivo biodistribution and PET-CT imaging of [^64^Cu]Cu-TE1PA-9E7.4 in normal (healthy) and tumour-bearing mice. (**A**) ex vivo biodistribution of [^64^Cu]Cu-TE1PA-9E7.4 in normal mice and (**B**) MOPC315.BM tumour-bearing mice at 5 min, and 2, 24, 48 and 72 h after intravenous injection of 8.5 ± 0.6 MBq of [^64^Cu]Cu-TE1PA-9E7.4. Values are expressed in %ID/g ± SD, (*n* = 4/group; *n* = 5 for 72 h p.i groups) (multiple *t* test, (*) *p* = 0.00001, (**) *p* = 0.000007, (***) *p* < 0.000001). (**C**). For illustration proposes, PET-CT images of a MOPC315.BM-tumour-bearing mouse at 5 min and 2, 24, 48 and 72 h after intravenous injection of 10,01 MBq of [^64^Cu]Cu-TE1PA-9E7.4. Images are presented as maximum intensity projection (MIP) and values are expressed in Bq/mL) and no smoothing was applied to PET and CT.

Comparative ex vivo biodistribution experiments were conducted in healthy mice (Figure 3A, Table S2) and subcutaneous (s.c) MOPC315.BM tumour-bearing mice (Figure 3B, Table S3). Relatively similar distribution kinetic profiles of [^64^Cu]Cu-TE1PA-9E7.4 were found for several organs in both models. [^64^Cu]Cu-TE1PA-9E7.4 exhibited rapid blood clearance with a maximum %ID/g in blood at 5 min post-injection (p.i) which then decreased rapidly at 2 h p.i (from 26.6 ± 7.8 %ID/g to 14.6 ± 8.2 %ID/g for healthy mice and from 27.7 ± 2.9 %ID/g to 10.9 ± 0.4 %ID/g for tumour-bearing mice). Same blood kinetics profiles were found in highly vascularised organs such as the heart, lungs and liver. In healthy mice, the higher uptake was found in the liver from 5 min p.i (35.8 ± 8.4 %ID/g) to 72 h p.i (15.7 ± 1.4 %ID/g). This uptake can be explained by the strong cell surface expression of syndecan-1 in normal hepatocytes [27,28]. A relatively high uptake was also detected in normal tissues such as the spleen (13.7 ± 2.8 %ID/g at 24 h p.i) and kidneys (8.8 ± 1.5 %ID/g at 24 h p.i) whereas a very low signal was observed in muscle at 24 h p.i (1.0± 0.3 %ID/g).

In MOPC315.BM s.c tumour-bearing mice (Figure 3B), the distribution profile of [^64^Cu]Cu-TE1PA-9E7.4 was similar in each organ with lower %ID/g, such as the liver at 24 h p.i., with 21.8 ± 5.3 %ID/g in tumour-free mice versus 10.4 ± 2.1 %ID/g in tumour-bearing mice. Tumour uptake was about 9.2 ± 0.7 %ID/g at 5 min p.i, then increased rapidly to 45.6 ± 5.1 %ID/g at 2 h p.i (*p* < 0.000001) and remained stable over time up to 72 h p.i. Remarkably, at 48 h p.i, a tumour uptake of 62.5 ± 17.1 %ID/g was observed indicating a favourable tumour-to-liver ratio of 6.8. 

Additionally, longitudinal small-animal PET-CT imaging was performed in female MOPC315.BM s.c tumour-bearing mice (Figure 3C) eleven days after cell injection. At the earliest time-point (5 min p.i), the localisation of the radiotracer was mainly vascular, with an important signal found in the highly vascularised organs, such as the heart, liver, spleen and kidneys up to 2 h p.i. At 24 h p.i. a high radioactive signal persisted in the tumour as well as in the liver while no signal was observed in the non-specific organs. Signal in the tumour and in the liver was considerably reduced but still visible until 72 h p.i. PET-CT images performed at 24 h post injection of [^64^Cu]Cu-TE1PA-IgG2a,κ (Figure S1) in tumour-bearing mice did not show any specific tumour uptake, confirming the specificity of [^64^Cu]Cu-TE1PA-9E7.4. At 24 h post-injection, hepatic uptake was intense and equivalent for both radioimmunoconjugates.

### 3.5. Dosimetry

**Figure 4:**
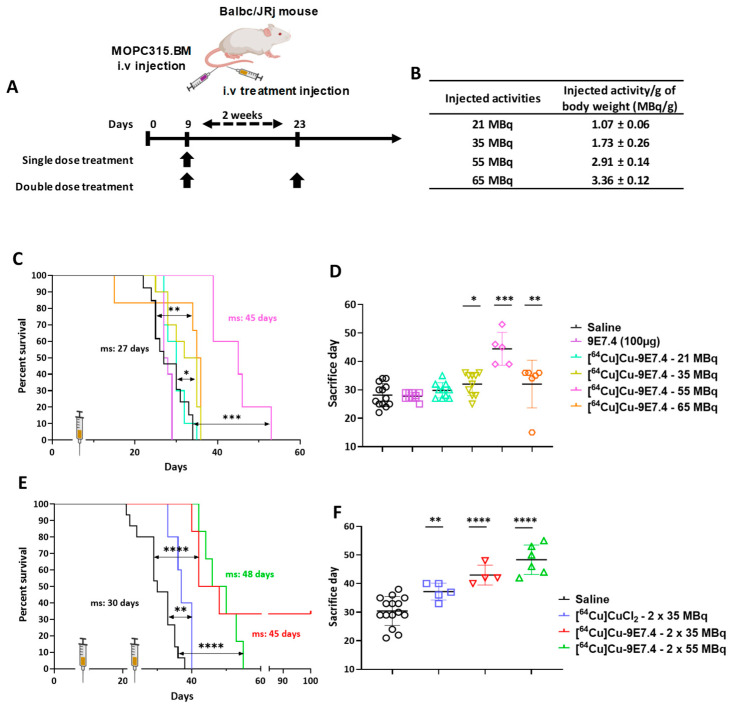
Activity-escalation study and repeated dose regimen. (**A**) Treatment schedule for single or repeated dose treatment after intravenous tumour cells injection (2 × 10^5^ cells). (**B**) Total activity injected per group expressed in MBq and MBq per gram of body weight (MBq/g). Kaplan–Meier survival curves of mice following single dose of treatment (**C**), and repeated doses of treatment (**E**) (*n* = 5–15; (*) *p <* 0.01, (**) *p* < 0.03, (***) *p* < 0.0002, (****) *p* < 0.0001; Log-rank (Mantel–Cox) test). Total duration of the study was 100 days. (**D**,**F**) Day of sacrifice of each mouse, bar represents the median survival, ^(1)^ surviving mice were excluded. ms: median survival. Created with Biorender.com.

Dosimetric calculations for a single injection of [^64^Cu]Cu-TE1PA-9E7.4 were performed based on ex vivo biodistribution data for [^64^Cu]Cu-TE1PA-9E7.4 (Figure 4). The average absorbed doses per MBq injected or for a single injection of 37 MBq are summarised in Table 1. Results show that the organs or tissues that received the highest absorbed doses were, in descending order, the tumour with 0.6 ± 0.1 Gy/MBq, the liver with 0.2 ± 0.0 Gy/MBq, the spleen with 0.1 ± 0.0 Gy/MBq and the kidneys with 0.1 ± 0.0 Gy/MBq. In all other healthy organs, the average absorbed dose was equal to or less than 0.05 Gy/MBq. Although the average absorbed dose to the liver appears to be significant, the tumour-to-liver absorbed dose ratio was 2.9, which appears to be favourable for the use of [^64^Cu]Cu-TE1PA-9E7.4 as a therapeutic agent.

### 3.6. Therapy Experiments on Orthotopic Model

The targeted radionuclide experiment started with a dose-escalation using a single injection of [^64^Cu]Cu-TE1PA-9E7.4 into the orthotopic model (cells injected intravenously) (Figure 4A). Treatments using non-radiolabelled 9E7.4 mAb alone or 21 MBq of [^64^Cu]Cu-TE1PA-9E7.4 did not improve median survival (27.5 days and 30 days respectively) compared with the saline control (27 days, *p* = 0.3251 and *p* = 0.4375 respectively) and all animals in these groups developed paraplegia (signs of disease) and extramedullary tumour masses revealed at autopsy. 

A relative but significant increase in survival compared with the control groups was achieved with a single injection of 35 MBq (ms = 33.5 days, *p* = 0.011) and treatment with a single administration of 55 MBq extended the median survival at 45 days (*p* < 0.0001). However, despite improved survival, animals in this group also developed hind leg paralysis. On the other hand, mice treated with the highest activity (65 MBq) showed a lower median survival (35.5 days) than those treated with 55 MBq (*p* = 0.0024). All animals of the 65 MBq group were sacrificed due to paralysis, excepted one mouse that was sacrificed prematurely after losing 20% of its initial body mass. This mouse received 3.6 MBq/g, which is higher than the average activity (3.4 ± 0.1 MBq/g of body weight) received by the other mice in this group (Figure 4B). Thus, the improvement in mouse survival appears to be activity dependent up to 55 MBq, after which it decreased (Figure 4D).

Based on the results obtained with activity-escalation, a repeated dose regimen was implemented using the two doses that showed better median toxicity-free survival. Effective activities of 35 and 55 MBq were selected for a repeat dose trial with a 2-week inter-dose interval (Figure 4C). While a single injection of 35 MBq increased the survival of mice by only 5.5 days compared with the saline group, the repeated injection of 35 MBq of [^64^Cu]Cu-TE1PA-9E7.4 significantly increased the median survival (*p* < 0.0001) of mice by 15 days (ms = 45 days) compared with the saline group (ms = 30 days). Moreover, two mice of this group (*n* = 6) were still alive at the end of the study (100 days). Although repeated dosing was beneficial with 35 MBq, repeated dosing of 55 MBq of [^64^Cu]Cu-TE1PA-9E7.4 did not significantly increase survival (*p* = 0.3217) compared with a single 55 MBq injection. It was, however, not less effective on survival than a single dose, and it increased median survival by 18 days compared with the saline group.

Effectiveness of copper-64 vectorisation was also evaluated by comparing the effect of the [^64^Cu]Cu-TE1PA-9E7.4-based therapy with two injections of 35 MBq of [^64^Cu]CuCl_2_. [^64^Cu]CuCl_2_, which was able to significantly increase median survival (*p* = 0.0096) by 7 days compared with the saline group. 

**Figure 5:**
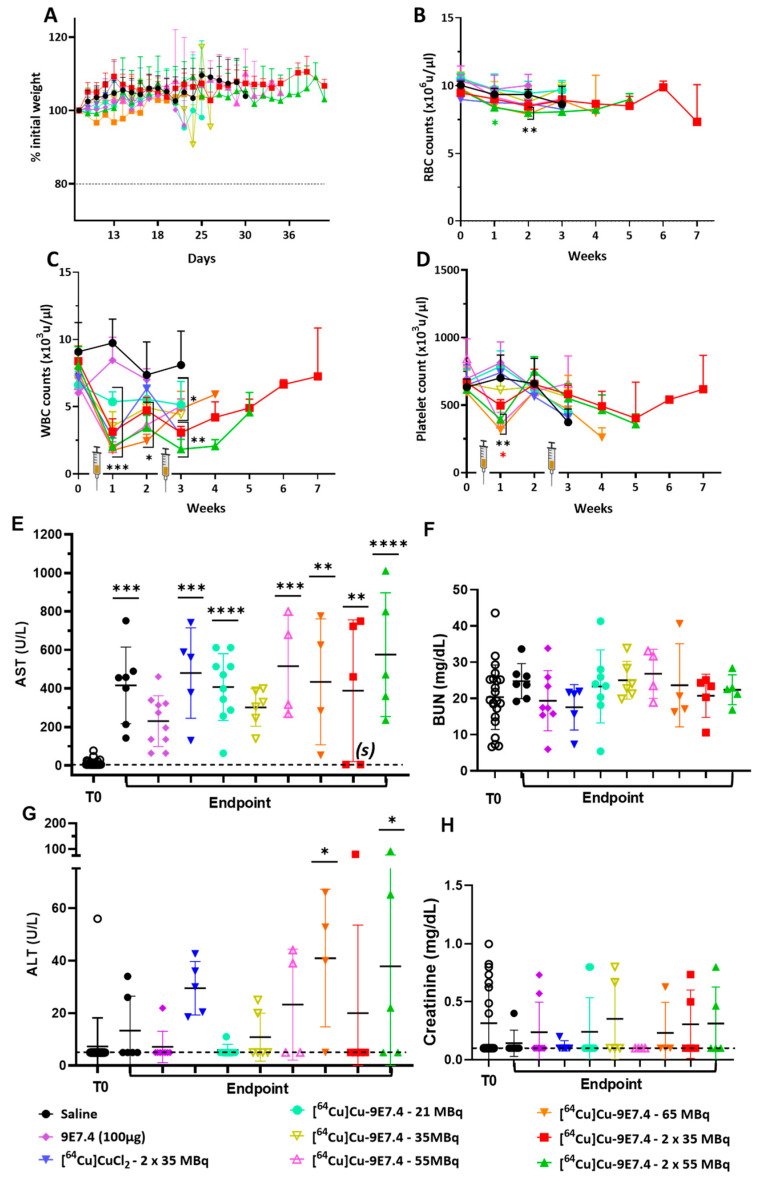
General and haematotoxicity (*n* = 5–26; BALB/cJRj mice) and biochemical toxicity (*n* = 4–22 BALB/cJRj mice) before and during therapy fellow-up. (**A**) Mean body weight variation expressed as a % of initial weight in each group. Haematotoxicity was assessed by counting (**B**) red blood cells, (**C**) white blood cells and (**D**) platelets once a week after MOPC315.BM injection in the tail vein. Plasma counting of (**E**) AST, (**F**) BUN, (**G**) ALT and (**H**) creatinine were assessed before tumour cells inoculation (T0) and at endpoint. Statistical analysis was carried out with 1-way ANOVA and Sidak’s multiple comparisons tests (statistical results shown are significantly different from T0, (*) *p* < 0.03; (**) *p* < 0.0007; (***) *p* < 0.0003; (****) *p* < 0.0001). AST: aspartate aminotransferase; ALT: alanine transaminase; BUN: blood urea nitrogen. (s) represents the values of the surviving mice.

General and haematological toxicities were investigated during the therapy follow-up. No significant loss in body weight was observed in any of the groups of mice (Figure 5A). Haematological toxicity evaluation was achieved by counting red blood cells, white blood cells and platelets once a week after tumour cells inoculation in different animal groups. No significant difference was observed in all groups for RBC count (Figure 5B). A significant but transient decrease in the number of white blood cells was observed the week following injection in all groups that received radioactive products (Figure 5C), followed by an increase 2 weeks after injection. For platelets, there was a significant and transient decrease of their number, but only in mice that received activities greater than 35 MBq (Figure 5D).

We also investigated liver and kidney toxicity by measuring aspartate aminotransferase (AST) and alanine transaminase (ALT) rates for liver toxicity and blood urea nitrogen (BUN) and creatinine for kidney toxicity before tumour cells inoculation (T0) and at sacrifice (Figure 5). No significant increase was observed for BUN or creatinine (*p* < 0.9999) after each treatment condition (Figure 5F–H). Regarding liver toxicity, AST levels at sacrifice were significantly increased compared with baseline levels except for groups injected with non-radiolabelled 9E7.4 or 35 MBq of [^64^Cu]Cu-TE1PA-9E7.4 (Figure 5E). It should be noted that, for the mouse sacrificed prematurely for excessive weight loss (treated with 65 MBq), AST level was relatively low while the surviving mice, treated with 2 × 35 MBq of [^64^Cu]Cu-TE1PA-9E7.4, had similar AST levels as mice at T0 (values (s) on the graph). Only the 65 MBq and 2 × 55 MBq [^64^Cu]Cu-TE1PA-9E7.4 groups of mice had significantly higher ALT levels (*p =* 0.0295 and *p =* 0.0310, respectively) (Figure 5G) than at T0, indicating liver damage. 

### 3.7. Therapy Experiments in Subcutaneous Model

**Figure 6:**
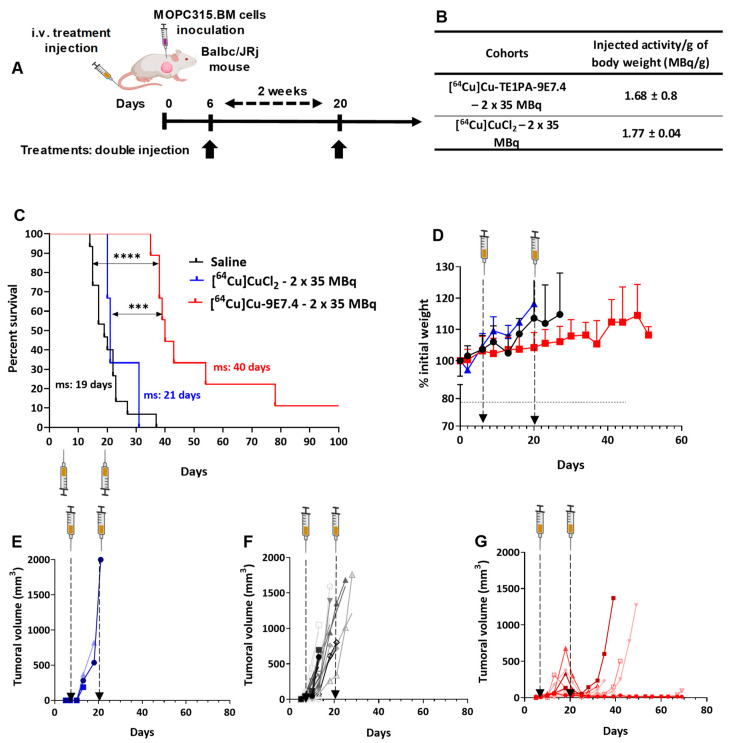
Therapy experiment with repeated dose of treatments and saline in BALB/cJRj mice bearing s.c MOPC315.BM tumours. (**A**) Treatment schedule for repeated dose treatment after s.c tumour cells injection (2 × 10^5^ cells). (**B**) Total activity injected per group expressed in MBq and MBq per gram of body weight (MBq/g). (**C**) Kaplan–Meier survival curves. Total duration of the study was 100 days. (*n* = 3–15; (***) *p <* 0.0002, (****) *p <* 0.0001, Log-rank (Mantel–Cox) test). (**D**) Mean body weight variation for each group expressed as a % of initial body weight. Tumour growth curves for mice receiving (**E**) [^64^Cu]CuCl_2_ (*n* = 3), (**F**) saline (*n* = 10), or (**G**) [^64^Cu]Cu-TE1PA-9E7.4 (*n* = 10). Each curve represents a mouse. Tumour volume was measured twice per week until signs of tumour necrosis, paralysis or volume reached 2000 mm^3^. Created with Biorender.com.

Six days after MOPC315.BM s.c inoculation (tumours were palpable), mice received the first intravenous injection of treatment (35 MBq of [^64^Cu]Cu-TE1PA-9E7.4, [^64^Cu]CuCl_2_ or 0.9% NaCl) followed by a second injection two weeks later (Figure 6A). 

Survival (Figure 6B) and tumour growth (Figure 6B) were monitored to evaluate treatment efficacy. Unlike in the orthotopic model, [^64^Cu]CuCl_2_ did not increase survival (ms = 21 days) compared with the saline group (ms = 19 days) (*p =* 0.5448) whereas survival was significantly increased, doubling the median survival (ms = 40 days) (*p <* 0.0001) for mice that had received 2 doses of [^64^Cu]Cu-TE1PA-9E7.4. Two mice receiving this treatment survived up to 80 days post cell inoculation before disease recurrence in one case, while the other survived until the end of the study. 

Body weight follow-up during the experiment (Figure 6C) did not reveal any significant weight loss in either the radioactive or saline treated groups. Concerning tumour growth, the anti-tumour effect of [^64^Cu]Cu-TE1PA-9E7.4 has been demonstrated (Figure 6F). In the first stage, injection of the radiolabelled antibody retarded tumour growth compared with the [^64^Cu]CuCl_2_ and saline injections (Figure 6D–F). After the first injection, tumours from the [^64^Cu]Cu-TE1PA-9E7.4-treated cohort had a significantly slower mean doubling time (dT) of 4.51 ± 2.17 days than the saline cohort (dT = 1.9 ± 0. (days, *p =* 0.012). At 18 days post cell inoculation, the average tumour volume was only about 195.1 mm^3^ for the mice treated with [^64^Cu]Cu-TE1PA-9E7.4 versus 925.7 mm^3^ for the saline group and 678.6 mm^3^ for the mice treated with [^64^Cu]CuCl_2_. This antitumour effect was significantly enhanced following the second injection of [^64^Cu]Cu-TE1PA-9E7.4 in all animals, and resulted in a maximum average reduction of 70.7 ± 21.3% in tumour volumes. This treatment resulted in tumour disappearance in one mouse, a tumour recurrence observed at day 70 post cell inoculation for another and at least 20 days of survival for other mice of the group where a small tumour volume was maintained (~15 mm^3^). Tumour recurrence induced hindlimb paralysis reflecting migration of MOPC315.BM tumour cells to the bone marrow and disease occurrence. 

**Figure 7:**
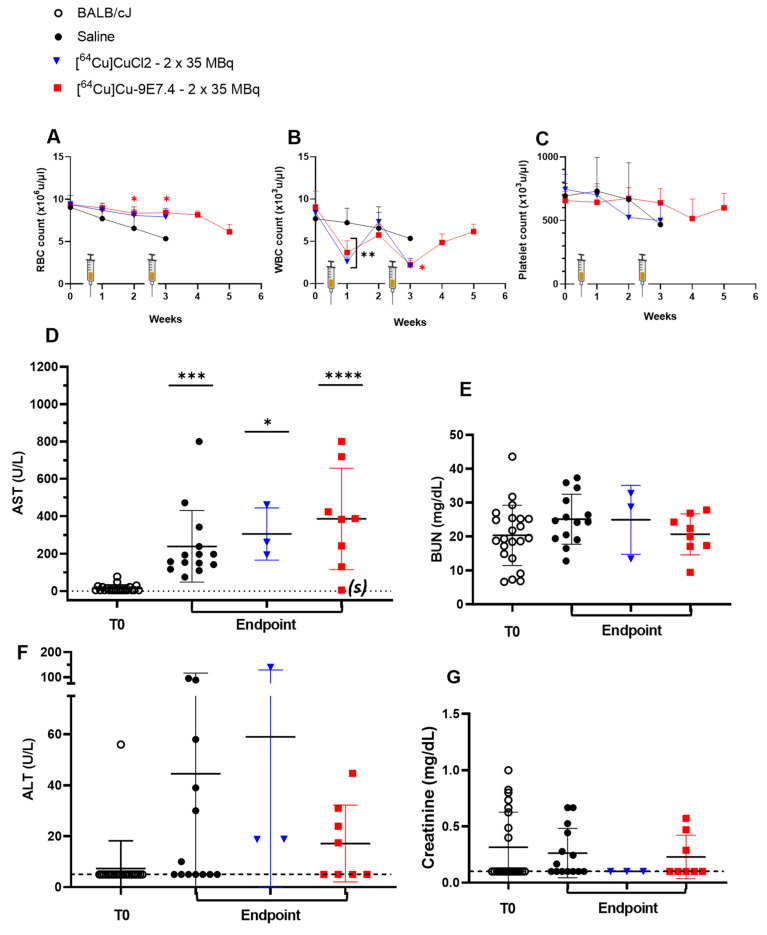
Haemotological and biochemical toxicity during therapy experiments in BALB/cJRj mice bearing s.c MOPC315.BM tumours (*n* = 3–22). Haematotoxicity was assessed by counting (**A**) red blood cells, (**B**) white blood cells and (**C**) platelets once a week after MOPC315.BM subcutaneous inoculation. Biochemical toxicity was evaluated by plasma counting of (**D**) AST, (**E**) BUN, (**F**) ALT, and (**G**) creatinine before tumour inoculation (T0) and at endpoint. Dashed lines represent the threshold values of parameters. Statistical analysis was carried out with 1-way ANOVA and Sidak’s multiple comparisons tests (statistical results shown are significantly different from T0, (*) *p* = 0.0237; (**) < 0.001; (***) *p* = 0.0007; (****) *p <* 0.0001). AST: aspartate aminotransferase; ALT: alanine transaminase; BUN: blood urea nitrogen.

As in the orthotopic model, injection of radioactivity induced a transient and moderate decrease in white blood cells count but had no effect on platelets and red blood cell rates (Figure 7). A steady decline over time in platelet, white blood cells and red blood cell counts induced by the progression of the disease is apparent in the saline control group. All of these results confirm the efficacy of this treatment regimen in controlling tumour growth and improving survival in this model. 

Biochemical analysis of liver toxicity showed a significant increase in mean AST levels in each of the three cohorts compared with the baseline levels observed at time T0 (Figure 7D), with a greater increase in values for the [^64^Cu]Cu-TE1PA-9E7.4-treated group (from 14.3 ± 12.7 U/L at T0 till 386.2 ± 196.2 U/L, *p* < 0.0001). Again, for the mouse that survived to the end of the study, the AST level assayed at sacrifice was equivalent to the baseline at T0 (see (s) on the graph). For ALT assays in each cohort at sacrifice, the values obtained were not significantly different than those of mice in the T0 reference group (Figure 7F). As in the orthotopic model, no significant differences were observed in creatinine (Figure 7G) or BUN levels (Figure 7E), which are markers of potential renal toxicity.

**Figure 8:**
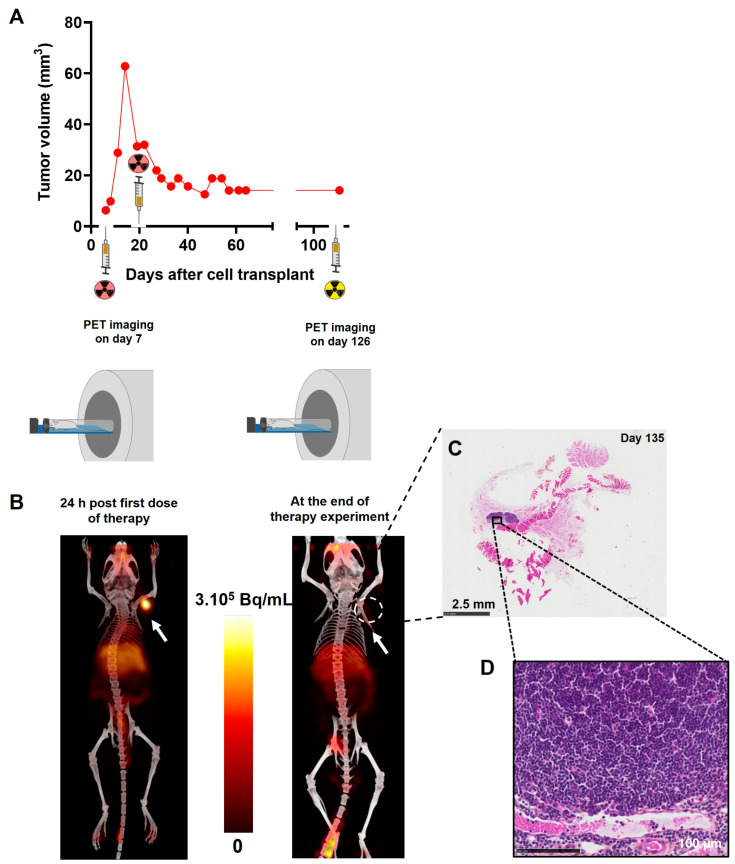
For illustration propose, specific results of the subcutaneous MOPC315.BM-bearing mouse that survived until the end of the study without any sign of disease or treatment-related toxicity are shown. (**A**) Tumour growth curve measured manually with a calliper. (**B**) PET-CT imaging performed at 24 h post injection of [^64^Cu]Cu-TE1PA-9E7.4 (29.6 MBq; 100 µg of 9E7.4 mAb) and at day 131 ([^64^Cu]Cu-TE1PA-9E7.4; 8,4 MBq; 25 µg of 9E7.4 mAb) post cells inoculation. Images are presented as maximum intensity projection (MIP) and no smoothing was applied to PET and CT images. Arrows indicate the location of MOPC315.BM tumour. (**C**) HPS histological staining of the tissue taken from the initial tumour site. (**D**) Magnification of histological staining at the lymph node.

To support the notion of [^64^Cu]Cu-TE1PA-9E7.4’s therapeutic efficacy, a precise follow-up of each of the mice was carried out, and results concerning the mouse that survived to the end of the study are presented in Figure 8. After the first injection of [^64^Cu]Cu-TE1PA-9E7.4, the tumour volume of this mouse increased slowly to a maximum volume of 62 mm^3^ 14 days after tumour transplantation (Figure 8A). TEP-CT imaging performed 24 h after the first therapeutic dose was injected confirmed the presence of the tumour, which was palpable, since the image reveals a strong signal at the tumour site (Figure 8B). At the second injection, the tumour volume measured by calliper was 31.4 mm^3^, the treatment reduced the tumour volume by 60% to 12.6 mm^3^ 46 days after tumour transplantation. After that, no recurrence of the tumour was observed in this mouse. To confirm complete recovery of this mouse, PET-CT imaging was performed 131 days post-inoculation and revealed no positive signal at the tumour injection site (Figure 8B). At sacrifice, autopsy revealed no visible tumour and tissue from the original tumour site was removed. Histological analysis of the tissue sample by HPS staining (Figure 8C) revealed muscle, connective tissue and a lymph node (Figure 8D). No evidence of residual tumour tissue was found locally.

## 4. Discussion

Theoretically, the nuclear properties of copper-64 (β^+^ 17.4%; β^−^ 39.0%, EC 44%) make it a promising radioisotope for theranostic use [2]. Indeed, copper-64 emits low energy positrons, which are suitable for PET imaging and allow one to obtain highly resolved PET images. Additionally, it emits β^−^ particles and Auger electrons enabling, via the adaptation of the administered activity, one to obtain a therapeutic effect. 

Here, we tested a theranostic approach using a ^64^Cu-radiolabelled antibody in an orthotopic and ectotopic murine model of multiple myeloma based on intravenous and subcutaneous injection of myeloma cells in immunocompetent mice. 

Based on the results previously published [20,26], we selected the TE1PA as a copper-64 chelating agent for the 9E7.4 antibody radiolabelling. Indeed, the use of the TE1PA chelate significantly reduces the transchelation phenomenon that causes the release of copper-64 in its free form and that can be taken up by the metalloproteins involved in copper transport. This transchelation phenomenon is more prominent when using DOTA or NOTA as copper-64 chelate and results in the redistribution of free copper-64 into the blood, lungs, and heart [20]. After radiolabelling and immunoreactivity verification, the radioimmunoconjugate, [^64^Cu]Cu-TE1PA-9E7.4, was tested for its potential theranostics performances including diagnostic and targeted radionuclide therapy.

First, the [^64^Cu]Cu-TE1PA-9E7.4 was evaluated as an imaging agent by PET-CT in subcutaneous tumour-bearing mice and ex vivo biodistribution at different times post radioimmunoconjugate injection. Contrasts were excellent at 24 h after [^64^Cu]Cu-TE1PA-9E7.4 injection, with high tumour-to-background ratios (Table S3). Ex vivo biodistribution studies were in accordance with radioactive signal in PET images, showing that tumour uptake of [^64^Cu]Cu-TE1PA-9E7.4 was 2.4-fold higher in the MOPC315.BM model, compared with the 5T33 model, with 32.3 ± 5.7 %ID/g and 13.6 ± 5.4 %ID/g, respectively [20]. This binding was significant and stable up to 48 h post injection. Hepatic uptake in this MOPC315.BM model was also high and was comparable with that found in the 5T33 model using [^64^Cu]Cu-TE1PA-9E7.4, with 9.1 ± 1.5 %ID/g and 12.0 ± 1.0 %ID/g respectively [20]. This high tracer uptake in the liver can be due to CD138 expression by normal hepatocytes that compose the liver parenchyma [28,29]. In all other tissues and organs, [^64^Cu]Cu-TE1PA-9E7.4 clearance was rapid, most notably in the vascular compartment which allowed identification of tumour foci as early as 2 h after tracer injection and then at 24 h with a good tumour-to-organ contrast. High contrast images are also due to the relatively low maximum energy of the positrons emitted by copper-64, which reduces the travel of the positrons in the matter before annihilation and limits the diffusion of the PET signal. 

The dosimetric study performed from the ex vivo biodistribution results was in line with PET-CT imaging. The long-lasting high tumour uptake appeared promising for therapy. However, according to dosimetric calculations, the liver was considered to be the critical organ in terms of toxicity, so particular attention was paid to the detection of toxicity signs in this organ during our therapeutic studies by measuring AST and ALT transaminases. 

Therapeutic experimentation on the orthotopic MOPC315.BM model demonstrated an anti-tumour effect of [^64^Cu]Cu-TE1PA-9E7.4 with significant improvement of survival in several cohorts. This efficacy cannot be attributed to the 9E7.4, as, when it was injected (as non-radiolabelled) at the same dose (100 μg), it did not increase mice survival and non-vectorized copper (^64^Cu) chloride ([^64^Cu]CuCl_2_) had only a very limited effect on survival. The potential efficacy of [^64^Cu]CuCl_2_ as a therapeutic agent is based on the elevated demand for copper from tumour cells and the overexpression of copper transports. In a preclinical study, [^64^Cu]CuCl_2_ was evaluated as a theranostic agent in B16F0 and A375M melanoma tumours, overexpressing the copper transporter Ctr1. The results show the ability of [^64^Cu]CuCl2 to visualise tumours by PET imaging and to produce an anti-tumour effect with a slowing cell proliferation and an improved survival of mice injected with 74 MBq compared with untreated mice [30].

In the orthotopic model, the maximum tolerated dose for [^64^Cu]Cu-TE1PA-9E7.4 was not reached in spite of elevated injected activities. Only skin toxicity at the injection site on the tail has been observed in some mice that had received the highest activities (65 MBq cohort). Dose-escalation showed that 55 MBq was the most effective activity in improving survival, but without cure. Similar results were obtained in the 5T33 model of MM with 18.5 MBq of [^177^Lu]Lu-DOTA-9E7.4 [13]. A recent preclinical study compared the therapeutic efficacy of copper-64 to a lutetium-177 human prostate cancer xenograft model [12]. The results show that a single injection of 150 MBq of [^64^Cu]Cu-DOTAH_2_-PSMA or 120 MBq of [^177^Lu]Lu-PSMA-617 significantly increased the median survival of mice compared with control mice with similar results and without inducing significant toxicity. However, these experiments were performed in immunodeficient mice with an NOD-*Rag1^null^ IL2rg^null^* (NRG) strain that is a radioresistant host [31]. Therefore, toxicity results need to be interpreted carefully within this particular context.

The therapeutic efficacy demonstrated in our study on immunocompetent mice was accompanied by moderate and transient haematologic toxicity affecting white blood cells and platelets. No platelet toxicity was observed in the MOPC315.BM heterotopic model. This seems to indicate an induced toxicity at the level of platelet precursors present in the BM where the tumour cells targeted by the treatment are also found in the orthotopic model. Additionally, in control mice, a progressive decrease in platelets over time was observed, indicating a possible toxicity induced by the progression of the disease. 

Beyond an activity of 55 MBq as a single dose, the treatment is no longer as effective. A lack of therapeutic efficacy was demonstrated for mice treated with 65 MBq that developed signs of paralysis. Contrary to that which was observed in the other cohorts, mice that received 65 MBq activity showed a significant increase in ALT levels at the time of sacrifice, reflecting liver toxicity. As with the other cohorts, the toxicity on white blood cell and platelet counts was transient, although relatively more severe and slower to return to physiological levels. It is also important to note that, in this 65 MBq treated group, one mouse that showed signs of acute toxicity had to be sacrificed very early after rapid and significant weight loss. These results suggest that the therapeutic effectiveness of 65 MBq is limited by the induced toxicity. Again, equivalent results were observed with a 27.75 MBq injection of [^177^Lu]Lu-DOTA-9E7.4 [13], suggesting deleterious toxicity to the liver and bone marrow. This beta emitter-induced liver and bone marrow toxicity has also been seen in the clinic. In particular with [^177^Lu]Lu-J591 in the treatment of metastatic prostate cancer, which causes bone marrow toxicity in patients and leads to a limitation of administered activities [32]. 

In nuclear medicine, therapeutic protocols deliver the treatment by fraction in order to preserve healthy tissue as much as possible and to potentiate its effectiveness [33]. Previous preclinical studies have also demonstrated the efficacy of repeated dosing compared with single-dose therapy [34,35,36]. Therefore, we evaluated a repeat dose of [^64^Cu]Cu-TE1PA-9E7.4 in the preclinical orthotopic model. Monitoring of haematological toxicity during dose escalation allowed us to identify the nadir of the most treatment-impacted blood elements, white blood cells and platelets, which is reached one week after treatment injection. For repeated-dose therapy experiments, we chose a 2-week dose interval to allow the mice to recover from the effects of the first dose before treating again. 

Although our study in the orthotopic model was performed in small cohorts, repeat treatments with 35 MBq (*n* = 6) or 55 MBq (*n* = 6) of [^64^Cu]Cu-TE1PA-9E7.4 significantly improved the survival of mice compared with the control mice. Repeated administration of 35 MBq of [^64^Cu]Cu-TE1PA-9E7.4 resulted in the survival of two mice, or approximately 30% of the cohort, who showed no signs of toxicity until the end of the study. In these two mice, AST levels measured at sacrifice were equivalent to those of the mice before tumour cell engraftment. In parallel, AST levels increased in the diseased mice, both control and treated. The evolution of this transaminase level could not be regularly evaluated during the study, which seems predictive of the disease progression, and can serve as a prognostic factor. Clinical studies have previously been able to identify liver abnormalities in MM patients that presented increased AST levels [37], and another study has suggested that elevated AST levels in patients are associated with a poor vital prognosis [38]. Furthermore, the aggressiveness and robustness of the MOPC315.BM model—for which it has been shown that, with only 1000 cells injected into mice, 100% of individuals developed paraplegia within 3–4 weeks [18]—allows us to demonstrate the efficacy of our treatment on these cured mice that would have inevitably developed signs of disease had they not been treated. 

Repeat dosing with 55 MBq activity did not improve the therapeutic response induced by a single 55 MBq dose. As with the cohort treated with 65 MBq, at sacrifice, ALT levels suggested liver toxicity that offset therapeutic efficacy. Injection of a third dose could almost certainly have further improved the survival of the mice, while avoiding liver or blood toxicity. This was demonstrated in a previous preclinical study in a mouse xenograft model of melanoma [39] in which injection of 3 doses of 25 MBq of [^131^I]ICF15002, a small molecule targeting melanin expressed by pigmented melanoma cells, slowed tumour proliferation with a doubling time of 17.7 ± 0.4 days with 2 doses of 25 MBq to 26.5 ± 7.8 days. Due to the logistics of ^64^Cu production and radiolabelling, other dosing schemes with multiple injections and/or shorter dose intervals could not be considered in this work. However, with our experimental conditions, we demonstrated the theranostic potential of [^64^Cu]Cu-TE1PA-9E7.4 with therapeutic efficacy comparable to or even more effective than lutetium-177 in the 5T33 model [13]. 

Beta emitters remain interesting because they are more readily available and at a lower cost, and their therapeutic efficacy is proven. The properties of beta emitters could have an advantage especially in more advanced stages of the disease with larger tumour lesions in which the beta particles ranges would be of interest. 

In this work we also evaluated the theranostic potential of [^64^Cu]Cu-TE1PA-9E7.4, on larger and less accessible tumour lesions. MOPC315.BM cells were subcutaneously injected in the same mouse strain, BALB/cJRj. This model permits a direct estimation of the treatment effect on the tumour mass in order to evaluate a possible anti-tumour effect. A repeated dose delayed tumour proliferation by about 20 days and significantly improved the survival of the mice. In parallel, although the small number of animals in this cohort (*n* = 3) must be taken into account, the administration of [^64^Cu]CuCl_2_ failed to slow tumour progression. No survival improvement was observed compared with the control group. This lack of beneficial effect observed in the subcutaneous ectotopic model may be explained by the lack of accessibility of the tumour cells, whereas in the orthotopic model where the cells are injected intravenously, the myeloma lesions are close to the vascular zone, so that a relative therapeutic effect was observed.

With the injection of [^64^Cu]Cu-TE1PA-9E7.4 a moderate and transient decrease in white blood cells was observed, and, in two mice, the antiproliferative effect lasted for several weeks with one mouse being completely cured. A preclinical study evaluating the therapeutic potential of copper-64 on a primary tumour mass showed similar results in a rat pancreatic cancer model with a somatostatin-like peptide [7]. Repeat injections of 3 doses (3 × 740 MBq) stopped tumour proliferation for about 10 days during which the tumours were not palpable, before they grew back in all animals. 

Interestingly, in the cohort of subcutaneous tumour-bearing mice treated with [^64^Cu]Cu-TE1PA-9E7.4, the mice were killed before the end of the study due to the appearance of signs of lower limb paralysis. The control mice were sacrificed because of a tumour mass approaching 2000 mm^3^. It seems that, with the prolonged survival, the MOC315.BM cells had time to detach from the tumour mass and migrate to the bone marrow. This observation corroborates the healing results obtained in the orthotopic model. Indeed, in the mouse that survived without tumour recurrence for up to 80 days, the residual cells in the bone marrow after treatment eventually proliferated given the highly invasive nature of this tumour line. This explains the very late recurrence observed in this mouse. On the other hand, the cure seems complete for the mouse in which no signs of the disease were observed, even after 100 days.

Our approach combines the exceptional specificity/affinity of the 9E7.4 anti-CD138 antibody with the high sensitivity of ^64^Cu-based PET imaging. On the one hand, ^64^Cu allows rapid, stable and high molar activity radiolabelling of the antibody at room temperature and particular decay properties of ^64^Cu allows for high resolution PET images. On the other hand, the high affinity/specificity of the 9E7.4 antibody for CD138 and the sufficiently high and long-lasting tumour uptake of the radio-immunoconjugate allows for efficient irradiation of the tumour lesions.

Our results provide confirmation of the theranostic potential of ^64^Cu-radiolabelled vectors and pave the way for further theranostic evaluation of copper-64-based therapies, evaluating different therapeutic regimens including shorter treatment intervals or longer administration cycles, as well as combination therapy with chemotherapy or immunotherapy. A recent study has reported the enhanced effectiveness of combination therapy using [^64^Cu]Cu-NOTA-panitumumab F(a’) 2 with radiosensitizing Gemcitabine and the PARP inhibitor, rucaparib, in terms of slowing tumour growth and improving median survival [8]. Additionally, combination of ^64^Cu-based targeted radionuclide therapy using the αvβ3 integrin-targeted ^64^Cu-DOTA-EB-cRGDfK (925 MBq/kg) with programmed death-ligand 1 (PD-L1)-based immunotherapy using αPD-L1 mAb (10 mg/kg) has been tested in an MC38 tumour model and resulted in a significant delay in tumour growth [40]. It is well established that ionizing radiation-based therapies (radiotherapy and TRT) can stimulate the immune system in several ways. For example, targeted radiotherapy, including RIT, can contribute to the shifting of an immunosuppressive tumour microenvironment to a more favourable immune stimulatory effect. Hence, it makes sense to combine TRT with immunotherapy to substantially enhance efficacy.

## 5. Conclusions

In this work, we demonstrate the ability of the copper-64 radiolabelled monoclonal antibody 9E7.4, coupled with the chelate TE1PA, to detect tumour cells that express CD138 in the MOPC315.BM syngeneic murine model of MM. This detection performance, combined with the performance of copper-64 for PET imaging, allowed the [^64^Cu]Cu-TE1PA-9E7.4 to produce highly contrasted diagnostic PET images. Dosimetric estimation from ex vivo biodistribution data revealed acceptable absorbed dose values to healthy organs and tissues, and a very encouraging tumour absorbed dose for targeted radionuclide therapy applications. With high activities, the treatment showed therapeutic efficacy resulting in delayed tumour growth and increased survival without inducing major or irreversible toxicity. These promising results also demonstrate that repeated injections of [^64^Cu]Cu-TE1PA-9E7.4 are safe and can be effective in this syngeneic preclinical model of MM. 

## Figures and Tables

**Table 1:** Estimated absorbed doses to tissues/organs per MBq or for a single injection of 37 MBq from intravenous injection of [^64^Cu]Cu-TE1PA-9E7.4 in BALB/cJRj female mice bearing subcutaneous MOPC315.BM tumours.

Organ/Tissues	Mean Absorbed Dose (Gy/MBq) ± SD	Mean Absorbed Dose (Gy) ± SD for 37 MBq
Tumour	0.6 ± 0.1	21.3 ± 5.0
Liver	0.2 ± 0.0	7.4 ± 0.9
Spleen	0.1 ± 0.0	5.2 ± 0.5
Intestine wall	0.0 ± 0.0	1.5 ± 0.3
Kidneys	0.1 ± 0.0	4.5 ± 0.4
Ganglia	0.0 ± 0.0	0.5 ± 0.1
Lungs	0.0 ± 0.0	1.7 ± 0.4
Heart	0.0 ± 0.0	1.9 ± 0.2
Stomach wall	0.0 ± 0.0	1.2 ± 0.1
Salivary glands	0.0 ± 0.0	0.8 ± 0.1
Bone	0.0 ± 0.0	0.8 ± 0.2
Pancreas	0.0 ± 0.0	0.5 ± 0.0
Brain	0.0 ± 0.0	0.2 ± 0.0

## Data Availability

The data that support the findings of this study are available from the corresponding author, [LRV], upon reasonable request.

## Supplementary Materials

The following supporting information can be downloaded at: https://www.mdpi.com/article/10.3390/pharmaceutics15071817/s1, Table S1: Specific activity of [^64^Cu]Cu-TE1PA-9E7.4. Figure S1: In vivo PET-CT imaging in MOPC315.BM tumour-bearing mice; Table S2: Ex vivo biodistribution experiments in normal (healthy) mice. Table S3: Ex vivo biodistribution experiments in MOPC315.BM tumour-bearing mice.
