# Supplementary material for: Preclinical Evaluation of a 64Cu-Based Theranostic Approach in a Murine Model of Multiple Myeloma
Source: Pharmaceutics. 2023 Jun 25;15(7):1817. doi: 10.3390/pharmaceutics15071817 (PMC10385603; doi:10.3390/pharmaceutics15071817)
Supplement: Supplementary file 1 [file pharmaceutics-15-01817-s001.zip › pharmaceutics-2408354-supplementary.pdf]

pharmaceutics

MDPI

Supplementary Materials

Preclinical Evaluation of a ^{64}Cu-Based Theranostic Approach in a Murine Model of Multiple Myeloma

Cassandra Métivier [1], Patricia Le Saëc [1], Joëlle Gaschet [1], Catherine Chauvet [1], Séverine Marionneau-Lambot [2], Peter O. Hofgaard [3], Bjarne Bogen [3], Julie Pineau [4], Nathalie Le Bris [4], Raphaël Tripier [4], Cyrille Alliot [1,5], Férid Haddad [6,5], Michel Chérel [1,7], Nicolas Chouin [8], Alain Faivre-Chauvet [2] and Latifa Rbah-Vidal [1,*]

1 Nantes Université, Inserm, CNRS, Université d'Angers, CRCI²NA, 44007 Nantes, France; cassandra.metivier@etu.univ-nantes.fr (C.M.); patricia.lesaec@univ-nantes.fr (P.L.S.); joelle.gaschet@univ-nantes.fr (J.G.); catherine.chauvet@univ-nantes.fr (C.C.); cyrille.alliot@univ-nantes.fr (C.A.); michel.cherel@univ-nantes.fr (M.C.)
2 Nantes Université, Inserm, CNRS, Université d'Angers, CRCI²NA, CHU Nantes, 44007 Nantes, France; severine.marionneau-lambot@univ-nantes.fr (S.M.-L.); alain.faivre-chauvet@univ-nantes.fr (A.F.-C.)
3 Department of Immunology, Oslo University Hospital, 04024 Oslo, Norway; p.o.hofgaard@medisin.uio.no (P.O.H.); bjarne.bogen@medisin.uio.no (B.B.)
4 Univ. Brest, UMR CNRS 6521 CEMCA, 6 Avenue Victor le Gorgeu, 29200 Brest, France; julie.pineau@univ-brest.fr (J.P.); nathalie.lebris@univ-brest.fr (N.L.B.); raphael.tripier@univ-brest.fr (R.T.)
5 GIP ARRONAX, 44800 Saint-Herblain, France
6 IMT Atlantique, Nantes Université, Subatech, 44307 Nantes, France; haddad@arronax-nantes.fr
7 Nuclear Medicine Department, ICO-René Gauducheau Cancer Center, 44800 Saint-Herblain, France
8 Nantes Université, Inserm, CNRS, Université d'Angers, Oniris, CRCI²NA, 44007 Nantes, France; nicolas.chouin@oniris-nantes.fr
* Correspondence: latifa.rbah-vidal@univ-nantes.fr

Table S1 : Specific activity of [^{64}Cu]Cu-TE1PA-9E7.4.

Experiments	PET imaging	Therapy using 22MBq	Therapy using 35 MBq	Therapy using 55 MBq	Therapy using 65 MBq
Specific activity (MBq/mg) at the end of radiosynthesis	236	262	383 ± 46	580 ± 50	651

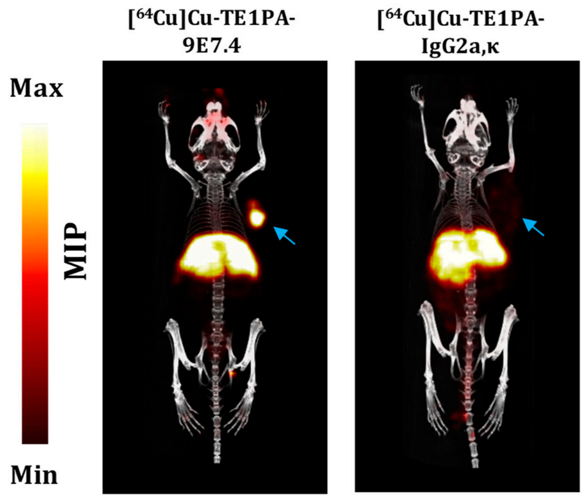

Figure S1. In vivo PET-CT imaging in MOPC315.BM tumour-bearing mice. Images were acquired 24 h after i.v injection of [^{64}Cu]Cu-TE1PA-9E7.4 (10.01 MBq ;25 µg of 9E7.4) or [^{64}Cu]Cu-TE1PA-IgG2a,κ (7.1 MBq; 25 µg of IgG2a,κ isotype). Mice were imaged 5 minutes, 2, 24, 48 and 72 h post injection. Images are presented as MIP ("maximum intensity projection"). Blue arrows indicate the location of the MOPC315.BM tumour.

Table S2: Ex vivo biodistribution experiments in normal (healthy) mice. Experiments were performed at 5 minutes, 2, 24, 48 and 72 h post intravenous injection of [^{64}Cu]Cu-TE1PA-9E7.4 (8.5 ± 0.75 MBq ;25 µg of 9E7.4).. * Values statistically different from the values obtained in the biodistribution of mice bearing a MOPC315.BM s.c tumour; † statistically different from that obtained at the previous time p.i (multiple t-tests with the Holm-Sidak method, p<0.05).

	**Healthy mice**				
	**5 min p.i**	**2 h p.i**	**24 h p.i**	**48 h p.i**	**72 h p.i**
**Liver**	35.8 ± 8.4	27.8 ± 12.3	21.8 ± 5.8 *	15.0 ± 3.8 *†	15.7 ± 1.4 *
**Blood**	26.6 ± 7.8	14.6 ± 8.2 †	7.0 ± 1.2 *	5.3 ± 0.6 *	2.7 ± 0.07 *
**Muscle**	1.1 ± 0.2	0.9 ± 0.2	1.0 ± 0.3	0.8 ± 0.1	0.9 ± 0.2
**Salivary glands**	3.3 ± 1.1	ND	3.8 ± 0.9	3.4 ± 0.3	3.1 ± 0.3 *
**Brain**	1.2 ± 0.4	0.8 ± 0.4	0.4 ± 0.0	0.3 ± 0.0	0.4 ± 0.1
**Nodes**	0.9 ± 0.2	0.8 ± 0.2	1.7 ± 0.4	1.5 ± 0.3	1.5 ± 0.2
**Bones**	2.1 ± 0.4	2.0 ± 1.0	1.9 ± 0.4	1.7 ± 0.4	1.5 ± 0.2
**Lungs**	11.0 ± 3.8	6.1 ± 3.1	6.5 ± 1.5	5.8 ± 1.2	5.8 ± 0.9 *
**Heart**	9.2 ± 3.1	5.1 ± 2.5	3.6 ± 0.8	3.0 ± 0.4	2.5 ± 0.2
**Spleen**	6.9 ± 1.7	10.9 ± 4.7	13.7 ± 2.7 *	12.5 ± 2.5 *	11.8 ± 1.4 *

**Kidneys**	9.8 ± 3.0	10.5 ± 4.7	8.8 ± 1.5	7.1 ± 0.8	5.2 ± 0.4 *
**Empty bowel**	3.0 ± 0.8	3.8 ± 1.1	7.5 ± 1.5 *	8.5 ± 1.3 *	6.9 ± 0.7 *
**Empty stomach**	1.7 ± 0.8	2.3 ± 0.4	3.5 ± 0.9	3.2 ± 0.8	3.2 ± 0.1 *

Table S3: Ex vivo biodistribution experiments in MOPC315.BM tumour bearing mice. Experiments were performed at 5 minutes, 2, 24, 48 and 72 h post intravenous injection of [^{64}Cu]Cu-TE1PA-9E7.4 (8.46 ± 0.47 MBq ;25 μg of 9E7.4). Tumor-to-selected tissues ratios at 24 and 48 h p.i. are also presented. * Values statistically different from the values obtained in the biodistribution of healthy mice; † values statistically different from that obtained at the previous p.i. time (multiple t-tests with the Holm-Sidak method, $p<0.05$).

	**Tumour-bearing mice s.c MOPC315.BM**				
	**5 min p.i**	**2 h p.i**	**24 h p.i**	**48 h p.i**	**72 h p.i**
**Tumor**	9.2 ± 0.7	45.6 ± 5.1 †	32.3 ± 5.7 †	62.5 ± 17.1 †	33.2 ± 12.5 †
**Liver**	29.8 ± 0.4	23.5 ± 1.6 †	10.4 ± 2.1 *†	9.1 ± 1.5 *†	7.2 ± 1.0 *
**Blood**	27.8 ± 2.9	10.9 ± 0.4 †	2.8 ± 0.3 *†	2.5 ± 0.3 *	1.4 ± 0.2 *
**Muscle**	0. 8 ± 0.1	0.7 ± 0.3	0.5 ± 0.1	0.4 ± 0.1	0.4 ± 0.0 *
**Salivary glands**	2.5 ± 0.4	1.9 ± 0.3	1.5 ± 0.1	1.6 ± 0.1	1.1 ± 0.1
**Brain**	1.5 ± 0.1	0.3 ± 0.1	0.2 ± 0.0	0.2 ± 0.0	0.2 ± 0.0
**Nodes**	0.8 ± 0.2	1.3 ± 1.3	1.7 ± 0.6	0.98 ± 0.2	1.0 ± 0.4
**Bones**	2.0 ± 0.3	1.6 ± 0.3	0.9 ± 0.1	1.0 ± 0.1	0.7 ± 0.1
**Lungs**	12.6 ± 2.4	5.5 ± 0.3 †	3.0 ± 0.3	3.1 ± 0.4	2.0 ± 0.3 *
**Heart**	8.9 ± 0.6	4.3 ± 0.3 †	1.6 ± 0.1	1.6 ± 0.1	1.3 ± 0.1
**Spleen**	5.3 ± 0.5	7.3 ± 1.2	4.9 ± 0.8 *	6.1 ± 0.8 *	4.3 ± 0.8 *
**Kidneys**	8.6 ± 0.7	12.0 ± 1.0	6.8 ± 0.2 †	6.1 ± 0.5	3.5 ± 0.1 *
**Empty bowel**	2.33 ± 0.37	4.9 ± 0.7	3.0 ± 0.5 *	4.0 ± 0.8 *	2.0 ± 0.3 *
**Empty stomach**	1.55 ± 0.18	2.2 ± 0.1	1.4 ± 0.1	1.8 ± 0.2	1.1 ± 0.1 *
Tumor-to-blood ratio			*11.5 ± 2.0*	*24.2 ± 5.4*	
Tumor-to-muscle ratio			*65.5 ± 22*	*145 ± 44*	
Tumor-to-liver ratio			*3.1 ± 0.1*	*6.7 ± 1.5*	
Tumor-to-bone ratio			*37.8 ± 7.9*	*62.2 ± 13.7*	
